# Survey of *C. difficile*-Specific Infection Control Policies in Local Long-Term Care Facilities

**DOI:** 10.4236/ijcm.2014.57056

**Published:** 2014-04-01

**Authors:** Laurie Archbald-Pannone

**Affiliations:** Department of Internal Medicine, Division of General Medicine, Geriatrics, and Palliative Care, Division of Infectious Diseases and International Health, University of Virginia, Charlottesville, USA

**Keywords:** *Clostridium difficile* Infection, Infection Control Policies, Long-Term Care Facilities

## Abstract

**Introduction:**

The incidence and severity of *Clostridium difficile* infection (CDI) has been increasing and long-term care facility (LTCF) residents are at high risk given their age, co-morbidities, and high antibiotic exposure. Infection control policies are crucial for controlling CDI, but there are currently no regulatory guidelines in the United States. Therefore, we evaluated infection control policies in local LTCFs to define the CDI-specific policies and the administrative and staff understanding of CDI, so as to identify perceived barriers for compliance.

**Methods:**

IRB approval was sought and exemption granted, all 8 local LTCFs were asked to participate. Each facility was visited by study personnel who interviewed the administrative Infection Control Practitioner (ICP) and 3 - 4 Licensed Practical Nurses (LPNs) with distinct survey format. Infection control policies were then compared to the SHEA recommendations for CDI in LTCFs.

**Results:**

Of the eligible facilities, 75% (n = 6) participated. ICP (n = 6) and LPNs (n = 21) were interviewed. All facilities accept residents with active CDI and 2 had written CDI-specific infection control policies. All facilities had hand hygiene or glove use policies and 2 had policies for the use of sporicidal environmental cleaning. No facility restricted antibiotic use. Each facility has a policy to instruct their staff through in-services, either annually or upon new hire, but 33% (n = 7) LPNs reported no facility-based CDI training. While 80% (n = 17) of LPNs felt comfortable with the facility CDI policies, only 11 accurately restated it. ICPs felt the most relevant barrier to staff compliance was time constraints (n = 4, 67%), however, LPNs felt it was limited knowledge (n = 10, 48%) and poor communication (n = 2, 10%).

**Discussion and Conclusions:**

With the increasing incidence and severity of CDI in LCTF, few of the facilities surveyed had CDI-specific policies. Despite CDI-specific training, there is a perceived knowledge and communication gap for staff caring for residents with CDI.

## 1. Introduction

*Clostridium difficile* (*C. difficile*) is an anaerobic, spore-forming bacterium that is one of the most common causes of antibiotic-associated diarrhea and the most common cause of antibiotic associated colitis [1]-[3]. The incidence of *C. difficile* infection (CDI) has doubled in the past decade. Since 2000, the rate of adults in the United States diagnosed with CDI has increased from 80.3 to 172.1/10,000 population [4]. A similar increased incidence has been shown in other recent studies [5]-[7]. More important, it is not just the mere number of patients with CDI that has increased, but the severity has also increased dramatically [8].

While the increase in CDI mortality can be seen in all age groups, it is most dramatic in the population over 60 years [4]. In our local hospital, we have also noted an increased severity with increasing age. In review of our local hospital billing data from 2008, 10% (45 of 457) of all inpatient deaths of elderly patients had a concurrent diagnosis of CDI. This trend has continued and highlights the importance of understanding CDI in elderly patients and the need to determine what factors can indicate a patient at increased risk of a poor outcome.

CDI, however, is not an infection exclusive to hospitalized patients. It has been reported that nearly half of all healthcare-onset cases of CDI occur in long-term care facilities (LTCFs) [9] [10]. In 1993, Simor, *et al*. found an 8% point prevalence of CDI among LTCF residents receiving antibiotics [11]. In 2003, Centers for Disease Control and Prevention in the United States published that the rates for CDI were mostly increasing in the elderly population and that nearly 2% of all patients transferred from acute-care hospitals to LTCFs had active CDI [12]. This reflects the “community” of infection, especially with high transfer rates of patients between acute and long-term care facilities. As our patients move in and out of the hospital, *C. difficile* often follows them. In our hospital in 2008, 6% of all inpatients and 16% of elderly inpatients were discharged to LTCFs. Of the inpatients with CDI, 18% of inpatients and nearly one third (28%) of elderly patients with CDI diagnosed during their hospitalization were discharged to LTCFs. With these high rates of transfer, it’s critical to look at CDI in LTCFs.

The full impact of CDI on residents in LTCFs is not fully understood. Residents of LTCFs have been estimated to have the highest incidence of diarrheal illness among adults living in the developed world [13]. A report of deaths from diarrheal illnesses from 1979-1987 found that more than half occurred in those older than 74 years [14]. There are multiple population factors that contribute to these high rates, including transfers between LTCFs and acute care facilities, large numbers of medically frail residents with incontinence and cognitive disorders, and limited infection control resources [15]. This increased risk also reflects the frail population that can acquire *C. difficile* from their environment and, in the setting of waning immunity and antibiotic use, develop symptomatic infection [16]. In 1990, Thomas, *et al*. reported that 33% of LTCFs residents had *C. difficile* in their stool after antibiotic treatment [16]. In 2007, Riggs, *et al*. reported that 51% of LTCFs residents were asymptomatic carriers of toxigenic *C. difficile*, in that they did not have overt diarrhea [17].

Our research group did an observational study to better understand the true incidence of diarrhea in local LTCFs, as compared to the reported incidence [18]. We found that while 11% of residents were reported to have diarrhea, on examination of the stool, >60% had diarrhea and 71% of these stool samples had a significant amount of intestinal inflammation.

In face of these high rates and the large impact of diarrhea in this vulnerable population, there is a need to control the spread of CDI in LTCFs. However, there are no regulatory guidelines that are enforceable for CDI-related infection control policies in LTCFs. Since *C. difficile* is a spore-forming organism, the control of the spores in the environment is critical to preventing the spread of infection in the environment. Alcohol-based hand sanitizers are generally not completely effective at removing the spore from the hands of people who come in contact with infected environments. Therefore, traditional hand-washing with soap and water for twenty seconds is a more reliable means of physically removing spores. Additionally, the *Clostridia* spores are not eradicated from a contaminated environment with routine cleaning solutions; instead, a sporicidal, 10% bleach solution is required.

In face of the changing epidemiology and complex nature of the organism, Simor *et al*. authored the Society for Hospital Epidemiology of America (SHEA) Position Paper evaluating CDI in LTCFs and stated recommendations for CDI-specific prevention and infection control (Table 1) [19]. For this research project, we surveyed the staff and administration in our local LTCFs to determine policies, knowledge, and attitudes toward CDI.

## 2. Methods

IRB approval was sought and exemption granted, all 8 local (Virginia, United States) LTCFs, as listed on the state Department of Health website were asked to participate in this study. All facilities were independent and none were governmental or associated with a hospital. The administrator and/or medical director of each facility was contacted by a member of the research team to discuss study participation. If a facility agreed to participate, a member of the research team interviewed the Infection Control Practitioner (ICP) to determine facility policy with a standardized survey. With a distinct in survey, 3 - 4 Licensed Practical Nurses (LPNs) at that facility were interviewed to determine their knowledge of CDI, facility policy, and behavior treating residents with CDI. Each survey included questions concerning the presence of CDI-specific infection control policy, as well as perceived barriers to implementation of such policies. The answers to survey questions were recorded by a member of the research team during the interview and listed in an Excel spreadsheet. General themes for open-ended responses were then categorized and by primary author.

## 3. Results

Six of 8 (75%) LTCFs agreed to participate (Table 2). The 2 facilities that declined did not differ significantly to the 6 that did participate. Six infection control practitioners and 21 licensed practical nurses were interviewed. All facilities surveyed had written infection control policies that included hand hygiene and all facilities accepted residents with CDI.

Two (33%) facilities had written CDI-specific infection control policies. All facilities reported that the staff should wear gloves for contact with residents with active CDI and reported that the staff should be educated as to the transmission and epidemiology of CDI. Half of the facilities (n = 3) recommended private rooms for residents with CDI. Only 2 facilities reported that they used sporicidal agents for the direct environment of a resident with CDI. No facility restricted antibiotic usage or implemented an antibiotic stewardship program.

All facilities instructed their staff on infection control policies via in-service discussions, 5 of which were at least annually and upon new hire. 19 of the 21 LPNs interviewed identified *C. difficile* as causing intestinal infection or diarrhea.

Most of the LPNs (n = 15) reported they had CDI-specific training; 9 (43%) in the past year and 6 (28%) unsure of the timing (Table 2). Conversely, one-third (n = 7) of the LPNs surveyed reported that they never had any facility-based training on CDI. Although 81% (n = 17) of LPNs reported feeling comfortable with their facility’s policy on CDI, 65% (11 of 17) correctly stated the protocol.

Although 71% (n = 15) of LPNs estimated ≥90% compliance with the CDI protocol at their facility, 67% (n = 14) wanted more training and education. ICPs reported perceived barriers for staff compliance with CDI-policies as lack of time (67%, n = 4), limited knowledge of the staff (50%, n = 3) and undefined barriers (33%, n = 2, Table 3). LPNs reported the main barriers to compliance with CDI-policies were limited knowledge (n = 10, 48%), lack of time (n = 5, 24%), and poor communication of facility policies (n = 2, 9.5%).

## 4. Discussion

In this study only one-third (n = 2) of our local LTCFs have a written infection control policy specific for CDI. Although CDI-specific training occurs at each LTCF, both ICPs and LPNs perceive a lack of knowledge as a barrier for LPNs in caring for patients with CDI. While the ICPs felt the biggest barrier to implementation was the staff’s lack of time, the majority of LPNs wanted more training on how to best care for these patients and more communication from administration on the facility policies.

With the increasing severity of CDI in LCTF, this study attempts to evaluate infection control policies in our local LTCF. We have found that, while each facility surveyed had detailed infection control policies, few had CDI-specific infection control policies. There is no national, regulatory guideline for management of CDI in LTCF in the United States. However, in face of the changing epidemiology and complex nature of the organism, Simor *et al*. published recommendations for CDI Prevention and Infection Control in LTCFs (Table 1) [19]. In our survey of local LTCF, no facility had a policy that completely followed the SHEA recommendations for CDI prevention in LTCFs. Most of these facilities focus on use of gloves, education of the staff, and isolation of patients with CDI or new-onset diarrhea. While these are important components of a policy to limit CDI, comprehensive and regulatory guidelines are needed to ensure consistent infection control in all facilities. Since the treatment of CDI is complicated and not always effective, preventing the spread of these bacteria, especially to the most vulnerable population, is critical, and infection control policies in LTCFs should focus on prevention [2] [8] [20].

This study is limited in widespread applicability by the small sample size and the defined geographic area of facilities surveyed. A larger study that examines more widespread LTCF policies in locations without government regulations is needed. Wilkinson, *et al*. published a recent Canadian study to evaluate CDI-related practices [20]. In this study, Wilkinson, *et al*. analyzed survey results from 593 Canadian LTCFs, representing all provinces and territories. They found that 76% of LTCFs had CDI-specific infection control policies. Although LTCFs were significantly less likely to submit a liquid stool specimen for CDI testing, as compared to acute care facilities (24% vs. 4%, p < 0.001), only 1% (n = 4) did not implement contact precautions for residents with confirmed CDI. Thirty-six percent (n = 173) of LTCFs had made a recent change in their infection control policy in response to a recent CDI outbreak, most commonly increasing surveillance. In contrast to our study, most (78%, n = 390) of LTCFs surveyed had CDI-specific infection control policies in place. However, similar to our study, very few (n = 116) facilities with CDI-specific policies had a program to control the use of antibiotics.

## 5. Conclusion

Our study suggests that long-term care facilities not only consider the development of CDI-specific infection control policies, but also focus on the appropriate education and communication with the nursing staff to ensure facility compliance.

## Figures and Tables

**Table 1:** SHEA recommendations for CDI prevention and control in LTCFs.

Recommendation	Evidence
Healthcare providers should wear gloves for contact with LTCF residents with CDI their body substances and environment.	A, I
Use disposable, single-use thermometers.	A, II
Implement policies in the LTCF for the prudent use of antimicrobial agents.	A, II
Disinfection of the environment of a resident with CDI using sporicidal agents.	B, II
Surveillance of antimicrobial utilization in the facility.	B, III
Educate healthcare providers in the facility about the clinical features, transmission, and epidemiology of CDI.	B, III
Care for LTCF residents with CDI in a private room, if possible, until the diarrhea resolves.	B, III
Meticulous hand hygiene with soap or an antimicrobial agent is after contact with residents, body substances, or potentially contaminated environment.	B, III
Dedicated patient care items and equipment for residents with. If such items must be shared, they should be cleaned between residents.	B, III
Residents with CDI may be removed from isolation when their diarrhea has resolved.	B, III

**Table 2:** Survey results from infection control practitioner and licensed practical nurses in long-term care facilities.

**Facility (n = 6 of 8)**	**Number (%)**

Written infection control policy	6 (100)
CDI-specific infection control policies	2 (33)
Glove use for residents with active CDI	6 (100)
Staff should be educated on transmission and epidemiology of CDI	6 (100)
Recommend private rooms for residents with active CDI	3 (50)
Use of sporicidal agents for room of resident with active CDI	2 (33)
Restrict antibiotic use	0 (0)
Antibiotic stewardship program in facility	0 (0)
Staff educated on infection control policies by in-service discussions	6 (100)
Staff educated on infection control policies annually or upon new hire	5 (83)

**Staff Perceptions (n = 21)**	**Number (%)**

Knew *C. difficile* caused intestinal infection or diarrhea	19 (90)
Received facility based CDI-specific training (any time)	15 (71)
Within the past year	9 (43)
Unsure of timing	6 (28)
Never received facility based CDI-specific training	7 (33)
Felt comfortable with facility’s CDI policy	17 (81)
Correctly stated facility CDI policy	11 of 17 (65)
>90% compliance with facility’s CDI protocol	15 (71)
Desired more training and education on CDI and policies	14 (67)

**Table 3:** Perceived barriers for staff compliance to facility infection control and CDI policies.

Perceived barriers	ICP	LPN
Lack of time	4 (67)	5 (24)
Limited knowledge	3 (50)	10 (48)
Undefined	2 (33)	0 (0)
Poor communication	0 (0)	2 (10)
